# Circular RNA ROCK1, a novel circRNA, suppresses osteosarcoma proliferation and migration via altering the miR-532-5p/PTEN axis

**DOI:** 10.1038/s12276-022-00806-z

**Published:** 2022-07-25

**Authors:** Yize Liu, Guanzhen Qiu, Yinzhou Luo, Shanshan Li, Yeqiu Xu, Yuanzhuang Zhang, Jiayuan Hu, Peifeng Li, Hai Pan, Yong Wang

**Affiliations:** 1 Department of Orthopedic Surgery, The First Affiliated Hospital, College of Medicine, Zhejiang University, Zhejiang, 310003 Hangzhou, China; 2 Fourth Department of Orthopedics, Central Hospital Affiliated to Shenyang Medical College, Shenyang, People’s Republic of China; 3 Department of Respiratory, Central Hospital Affiliated to Shenyang Medical College, Shenyang, People’s Republic of China; 4 Department of Electrodiagnosis, Central Hospital Affiliated to Shenyang Medical College, Shenyang, People’s Republic of China; 5 Center for Precise Medicine, Shengyang Medical College, 110034 Shenyang, China; 6 Department of Neurosurgery and Dean’s Office, Central Hospital Affiliated to Shenyang Medical College, Shenyang, People’s Republic of China; 7 Central Laboratory, Central Hospital Affiliated to Shenyang Medical College, Shenyang, People’s Republic of China

**Keywords:** Bone cancer, Cell invasion, miRNAs

## Abstract

As the most prevalent bone tumor in children and adolescents, the pathogenesis and metastasis of osteosarcoma (OS) remain largely unclear. Here, we investigated the expression and function of a novel circular RNA (circRNA), circROCK1-E3/E4, which is back-spliced from exons 3 and 4 of Rho-associated coiled-coil containing protein kinase 1 (ROCK1) in OS. We found that circROCK1-E3/E4, regulated by the well-known RNA-binding protein quaking (QKI), was downregulated in OS and correlated with unfavorable clinical features of patients with OS. Functional proliferation and cell motility assays indicated that circROCK1-E3/E4 serves as a tumor suppressor in OS cells. Mechanistically, circROCK1-E3/E4 suppressed proliferation and migration by upregulating phosphatase and tensin homolog (PTEN) through microRNA-532-5p (miR-532-5p) sponging. In the constructed nude mouse model, circROCK1-E3/E4 inhibited tumor growth and lung metastasis in vivo. This study demonstrates the functions and molecular mechanisms of circROCK1-E3/E4 in the progression of OS. These findings may identify novel targets for the molecular therapy of OS.

## Bone cancer: Role of circular RNA explored

Understanding the role of a circular RNA molecule in bone cancer may provide a foundation for potential therapies. The factors underlying the development and progression of osteosarcoma, an aggressive bone cancer most common in young people, remain unclear. Circular RNAs (circRNAs), derived from RNA splicing events, have multiple functions in diseases such as cancer. Yong Wang at Zhejiang University, China, and co-workers had already demonstrated that the *ROCK1* gene plays critical roles in osteosarcoma progression. Now, they have identified a novel circRNA called circROCK1-E3/E4 derived from *ROCK1*, which acts as a tumor suppressor in osteosarcoma. In experiments on human cell lines and mouse models, they found that circROCK1-E3/E4 regulates a key pathway that suppresses the proliferation and migration of cancer cells. Poor prognosis is linked to downregulated levels of this circRNA.

## Introduction

Osteosarcoma (OS) is the most frequent malignant bone neoplasm, particularly among children and adolescents. The prediction site of osteosarcoma is most often in the metaphysis of long bones^1^. Owing to its aggressive characteristics, the prognosis of osteosarcoma is unfavorable. According to a previous study, the mortality rate of osteosarcoma was approximately 1.3% per year over the years 1990–2004 in the USA^2^. It was reported that approximately 20% of patients presented with pulmonary metastasis at their first visit due to the vicious biological behavior^2^. Consequently, it is compelling to seek out novel metastasis-related molecules and identify their underlying mechanism in osteosarcoma.

Circular RNAs (circRNAs), produced by precursor mRNA back-splicing of exons, are a distinguished kind of closed-ring structured transcripts without 5ʹ-end caps and 3ʹ-end poly A tails^3^. CircRNAs exhibit multiple functions, such as acting as miRNA sponges, interacting with proteins, being translated into proteins and regulating transcription^3^. Circular RNAs, serving as pseudogenes, regulate parental gene expression via a competitive endogenous RNA (ceRNA) mechanism^4–6^. CircRNAs are extensively implicated in various diseases, including osteosarcoma^7,8^. It has been reported that circular RNA TADA2A (circTADA2A), derived from exons 5 and 6 of TADA2A mRNA, increases CREB3-mediated malignant tumor behavior by readily sponging miR-203a-3p in osteosarcoma^9^.

Rho-associated coiled-coil containing kinase 1 (ROCK1) is a member of the AGC family of serine/threonine kinases, and ROCK1 was identified as an effector of RhoA^10,11^. ROCK1 has manifold functions in cancer-related pathogenesis, including cell contraction, migration, apoptosis, survival, and proliferation^12^. Previous studies from our group have also uncovered the oncogenic roles of ROCK1 in osteosarcoma^13–16^. According to Rybak-Wolf A and Salzman J’s study, a total of 53 circRNAs are derived from the human ROCK1 gene^17–19^. However, the expression and function of any ROCK1-back-spliced circRNAs in osteosarcoma remain largely unknown. In the present study, we concentrated on investigating the expression and function of hsa_circ_0108024, a circular RNA derived from exons 3 and 4 of the ROCK1 gene, in osteosarcoma. We found that hsa_circ_0108024, which we named circROCK1-E3/E4, serving as a tumor suppressor, inhibited proliferation and migration in osteosarcoma.

## Materials and methods

### Patients and tissue samples

A total of 50 osteosarcoma tissue specimens and paired paratumor tissue specimens were collected in Central Hospital Affiliated to Shenyang Medical College (Shenyang, China) and in Cancer Hospital of China Medical University/Liaoning Cancer Hospital & Institute (Shenyang, China) during surgical resections. The patients were clinically diagnosed with osteosarcoma according to a definite pathological diagnosis, and written informed consent was collected from these patients. Permission for this study was granted by the Institute Research Medical Ethics Committee of Central Hospital Affiliated to Shenyang Medical College.

### Cell culture

The human osteoblast cell line hFOB 1.19 was maintained in DMEM/F12 (Gibco, El Paso, TX, USA). Three human osteosarcoma cell lines, MG-63, U2OS, MNNG/HOS, 143B, purchased from the Chinese Academy of Sciences Cell Bank (Shanghai, China), were cultured in DMEM (Gibco). All cells were conditioned in a humidified atmosphere containing 5% CO_2_ at 37 °C. All media were supplemented with 10% (v/v) fetal bovine serum (FBS, Sigma, St. Louis, MO, USA), 100 IU/ml penicillin (Baomanbio, China) and 100 mg/ml streptomycin (Baomanbio, China).

### Actinomycin D assay and RNase R treatment

To determine the stability of circROCK1-E3/E4, an actinomycin D assay and an RNase R assay were performed. The actinomycin D assay was carried out as previously described^20^. In brief, 2 μg/ml actinomycin D (Merck KGaA) was added. Total RNA from different time points (0, 4, 8, 12, and 24 h) was extracted, and the expression of circROCK1-E3/E4 and linear ROCK1 mRNA was measured by reverse transcription quantitative polymerase chain reaction (RT–qPCR).

RNase R treatment was carried out as previously described^21^. Total RNA (2 mg) was incubated for 30 min at 37 °C with or without 5 U/μg RNase R (Epicenter Technologies Pvt. Ltd) and subsequently purified using an RNeasy MinElute Cleaning Kit (Qiagen, Inc.). Analysis was then performed by RT–qPCR.

### Nucleic acid electrophoresis

The cDNA and gDNA PCR products were investigated using 2% agarose gel electrophoresis with TAE running buffer. DNA was separated by electrophoresis at 120 V for 30 min. The DNA marker used was Super DNA Marker (CWBIO, Beijing, Cat. CW2583M). The bands were examined by UV irradiation.

### RNA-fluorescence in situ hybridization (FISH)

A FISH assay was employed to determine the subcellular localization of circROCK1-E3E4 in OS tissues and cell lines as previously described^22^. Paraffin-embedded OS tissues were sliced, dewaxed and gradient dehydration was performed. OS cell coverslips were rinsed and fixed with 4% paraformaldehyde at room temperature for 30 min. The tissue slices or cell coverslips were digested with 20 μg/ml proteinase K (Solarbio, Beijing, China) and washed three times with PBS for 5 min each. Hybridization buffer was added to tissue slices or cell coverslips and incubated at 37 °C for 1 h. One hour later, the prehybridization solution was removed, and the labeled fluorescent probes were added to the tissue slices or cell coverslips and incubated at 37 °C overnight. The next day, the tissue slices or cell coverslips were washed in a gradient with 2 × SSC (supplemented with 50% formamide, 5 min), 1 × SSC (5 min) and 0.5 × SSC (10 min) at 37 °C. The tissue slices or cell coverslips were incubated with 4’,6-diamidino-2-phenylindole (DAPI) for 8 min in the dark to counterstain nuclei, washed with PBS, air-dried, mounted, and photographed using a Leica SP5 confocal microscope (Leica Micosystems, Mannheim, Germany).

### RNA extraction, reverse transcription, and qPCR

The procedures were performed as previously described^23^. Total RNA was isolated using TRIzol reagent (Invitrogen, Carlsbad, CA, USA). For qualification of circRNAs, 3U RNase R enzyme per 1000 ng RNA was used to digest linear RNAs at 37 °C for 10 min. Isolated RNAs (circRNA, mRNA, and pre-mRNA) were reverse transcribed into cDNAs using a Goscript^TM^ Reverse Transcription System (Promega, Madison, WI, USA), and qPCR was carried out using GoTaq® qPCR Master Mix (Promega) according to the manufacturers’ instructions. GAPDH was used as an internal control for circRNAs and mRNAs, and U6 was used as an endogenous control for miRNAs. The relative expression levels of the genes were determined using the 2^–ΔΔCt^ method. The primer sequences are presented in Table 1.

**Table 1:** Primer and oligonucleotide sequences used in this study.

Gene	Primer (sense)	Primer (antisense)
hsa_circ_0047061	CTCTTCTTCAAAAATGACCAGTGGG	TTCTTCTACACCATTTCGCCCT
hsa_circ_0047060	AAATGGAGCAGAAGTGCAGAACC	CAAACTTTCCTGCTCTTCATCCAA
hsa_circ_0108012	GGAGCAGAAGTGCAGATACT	GCAAACTTTCCTGCAAGCTTTTAT
hsa_circ_0107991	GCTGCAACTGGAACTCAACC	TTAACAGCCGCTTGCATGTC
hsa_circ_0108010	GAGCAGAAGTGCAGAACCTCA	CTGCATTGGAGCTAGTTCTGT
hsa_circ_0108011	AAATCACGACATGGCCCCTC	CGTGGTGGCTCACACTTCTT
hsa_circ_0108016	AGCTTGCTGTAGCACCAGTT	AGGGAATGTTTCTTCCTCTCCTT
circROCK1-E3/E4	TGGGTTGTTCAGATAAAGACACA	TGCACCTCTACCAATCACCT
hsa_circ_0107997	AATGAAGGACAGATGCGGGAG	CTGCTCCAGTTGCAGGGTTAG
hsa_circ_0108001	GTGGCAATGTGTGCTCGAAT	AGCATGTCTTGAGCCTCTTTTC
hsa_circ_0047062	GCCTGAAAAATGGGCACGAT	ATGCCTTACCTGTGAATAAAACC
hsa_circ_0047056	GTTTTTAAGCCACCCCCTGC	CACATCTCCTTGGGTTACAGGT
hsa_circ_0107992	CCTTAAAACACAGAGAAACTCTTGC	TGTTCTTCCAGAAGGCCTCG
cANRIL	GCTGGGATTACAGGTGTGAGACACC	GAATCAGAATGAGGCTTATTCTTCTCATC
ROCK1 mRNA	AAGAGGGCATTGTCACAGCA	AGCATCCAATCCATCCAGCA
QKI mRNA	ACCTGCAGCAGAAGGAGAAG	AAGGCAAGGGCTGGTGATTT
PTEN mRNA	TCCCAGACATGACAGCCATC	TGCTTTGAATCCAAAAACCTTACT
GAPDH mRNA	AACGGATTTGGTCGTATTGGG	CCTGGAAGATGGTGATGGGAT
β-Actin mRNA	GATTCCTATGTGGGCGACGA	TGTAGAAGGTGTGGTGCCAG
ROCK1 intron 2	TAGTGGGCGCAGTTAGACAC	CCCTGTGGAAGAACCTTGCT
ROCK1 intron 4	TGAAGCTGTGGCCATGTTACT	CAAAACCCCACTTGACCCCA
miR-532-5p RT	GTCGTATCCAGTGCAGGGTCCGAGGTATTCGCACTGGATACGACACGGTC	
miR-532-5p	GCGGCGGCATGCCTTGAGTGTAG	ATCCAGTGCAGGGTCCGAGG
U6 RT	CGAGCACAGAATCGCTTCACGAATTTGCGTGTCAT	
U6	CGAGCACAGAATCGCTTCA	CTCGCTTCGGCAGCACATAT
**siRNA**	**Sense**	**Antisense**
siNC	UUCUCCGAACGUGUCACGU dTdT	ACGUGACACGUUCGGAGAA dTdT
sicircROCK1-1	GTTGTTCAGATAAAGACACAAUU	TTGTGTCTTTATCTGAACAACUU
sicircROCK1-2	GGTTGTTCAGATAAAGACACAUU	TGTGTCTTTATCTGAACAACCUU
siQKI-1	GCAUCUAAAUGAAGAUUUAUU	TAAATCTTCATTTAGATGCUU
siQKI-2	GGUACUUUAUGUAUAAUUAUU	TAATTATACATAAAGTACCUU
siPTEN-1	GAUGAUACAUGUAAAUUAAUU	TTAATTTACATGTATCATCUU
siPTEN-2	GAAUGGAUUUGAUACUUUAUU	TAAAGTATCAAATCCATTCUU
miR-532-5p mimic	CAUGCCUUGAGUGUAGGACCGU	GGUCCUACACUCAAGGCAUGUU
miR-532-5p inhibitors	ACGGUCCUACACUCAAGGCAUG	
miR NC	UUCUCCGAACGUGUCACGUTT	ACGUGACACGUUCGGAGAATT
Inh NC	CAGUACUUUUGUGUAGUACAA	

### Western blot analysis

The procedure was carried out as previously described^21^. In brief, proteins from cells or tissues were extracted by a total protein extraction kit (Abcam) and qualified by a BCA Protein Assay Kit (Abcam). Twenty micrograms of protein samples were subjected to electrophoresis, transferred onto a polyvinylidene difluoride membrane (EMD Millipore) and blocked with 5% skimmed milk for 60 min at room temperature. The membrane was incubated with primary antibody (Abcam) solution at 4 °C overnight, followed by 15 min of TBST washing three times. The membrane was incubated with secondary antibody at room temperature for 60 min. Signals of targeted proteins were detected using an enhanced chemiluminescence detection system.

### Plasmids, vectors, oligos, and cell transfection

Specific small-interfering RNAs (siRNAs) targeting circROCK1-E3/E4, QKI and PTEN and negative control siRNAs (siNC) were synthesized by GenePharma (Shanghai, China), and the specificities were qualified. To explore the formation of circROCK1-E3/E4, flanking intron segment 2 of ROCK1 (position of Chr18: 18649177–18649468, which we named FIS2), the sequence spanning exon 3 of ROCK1 (position of Chr18: 18666972-18667072) and the highly similar sequences in intron 4 of ROCK1 were cloned into pZW1-snoVector to obtain diverse targeted vectors (vectors #1, #2, #3 and #4, described in detail in the Results section) by Genscript (Nanjing, Jiang Su, China). Wild-type and mutant (with wild-type or mutated miR-532-5p response elements, MRE-532-5p) circROCK1-E3/E4 and PTEN overexpression plasmids were synthesized by GenScript. All plasmids, vectors, and oligos were transfected into U2OS and HOS cells according to the instructions of a Ribo TECT^TM^ Transfection Kit (166 T) (RiboBio, China). The related sequences of oligonucleotides are listed in Table 1.

### IHC and in situ hybridization (ISH)

Osteosarcoma tissues were fixed with 10% formalin, dehydrated via gradient, paraffin-embedded, sliced, dewaxed and rehydrated. Osteosarcoma Section (4-μm thick) were retrieved by antigen retrieval buffer, blocked with 3% hydrogen peroxide and sealed with 3% BSA. For IHC detection, the sections were incubated with primary antibodies (anti-QKI, cat. no. ab126742; anti-PTEN, cat. ab267787; Abcam, Cambridge, MA, UK) at 4 °C overnight. The next day, the sections were incubated with secondary antibodies (cat. no. ab6112, Abcam) for 30 min at room temperature, stained with 0.05% 3,3‑diaminobenzidine for 60 s, counterstained with 10% hematoxylin for 3 min, dehydrated with graded ethanol, and observed under a light microscope. For *ISH* detection, the sections were incubated with prehybridization solution for 1 h at 37 °C and then incubated with hybridization solution containing the miR-532 probe at 4 °C overnight. The next day, the sections were steeply washed with 2 × SSC (5 min, 37 °C), 1×SSC (5 min, 37 °C) and 0.5 × SSC (10 min, room temperature) and blocked with 5% BSA (30 min, room temperature). The sections were then incubated with mouse anti-digoxigenin-labeled peroxidase (anti-DIG-HRP) at 37 °C for 40 min. Then, the sections were stained with 0.05% 3,3‑diaminobenzidine for 60 s, counterstained with 10% hematoxylin for 3 min, dehydrated with graded ethanol, and observed under a light microscope.

### Cell counting kit-8 (CCK8) assay

The procedure was performed as previously described^16^. Briefly, HOS and U2OS cells with diverse interventions were incubated in 96-well plates (2 × 10^3^) supplemented with 200 µl culture medium and conditioned at 37 °C with 5% CO_2_. On Days 1, 2, 3, 4, and 5, 20 μl CCK-8 solution was added to each well and then incubated for 2 h. Absorbance was measured at an optical density of 450 nm using a microplate reader (Bio–Rad Laboratories, Inc.). Experiments were repeated in triplicate.

### 5‑Ethynyl‑20‑deoxyuridine (EdU) incorporation assay

The EdU incorporation assay was performed according to the protocol of a Click-iT® EdU Kit (Cat. no: C10338; Invitrogen, USA). HOS and U2OS cells (5 × 10^5^) with diverse interventions were seeded onto glass coverslips (18 × 18 mm) and cultured overnight. The next day, 1 ml of the medium was removed, an equal volume of EdU labeling solution (concentration of 20 µM) was added to each coverslip, and the coverslips were incubated under appropriate growth conditions. Two hours later, the culture medium was discarded, and the coverslips were fixed with 3.7% formaldehyde for 15 min at room temperature. The coverslips were washed twice with 1 ml of 3% BSA in PBS and then permeabilized with 0.5% Triton® X-100 for 20 min at room temperature. The permeabilization buffer was discarded, and the coverslips were washed twice with 3% BSA in PBS. The coverslips were incubated with 0.5 ml of Click-iT® reaction cocktail and rocked in the dark for 30 min at room temperature. The coverslips were washed with 3% BSA in PBS and labeled with 1× Hoechst® 3334 in the dark for 30 min at room temperature. The coverslips were observed under a fluorescence microscope (Leica, Wetzlar, Germany). The nucleus is blue after labeling with DAPI. Positive cells are presented in green. Images were analyzed using Image-Pro Plus software version 6.0 (Media Cybernetics, Inc., Rockville, MD, USA). The quantitative data are expressed as the percentage of EdU-positive nuclei relative to the total number of nuclei counted.

### Transwell assay

The procedure was performed as previously reported^15^. HOS and U2OS cells with diverse interventions (with an incubation density of 5 × 10^4^) were incubated in the upper chambers (Corning). Culture medium without and with 10% FBS was added to the upper and lower chambers, respectively. After 12 h, nonmigrated cells were wiped out, while migrated HOS and U2OS cells were fixed, stained and counted using an inverted microscope (Olympus Corporation).

### RNA immunoprecipitation (RIP) assay

The RIP assay was performed strictly according to the instructions of a Magna RIP RNA-Binding Protein Immunoprecipitation kit (EMD Millipore, USA). In brief, HOS and U2OS (2 × 10^7^) cells were harvested, and the cell pellet was lysed with RIP lysis buffer combined with a protease inhibitor cocktail and RNase inhibitor. Ten microliters of the supernatant of the cell lysate was used as the input. Magnetic beads were resuspended and vortexed twice with RIP wash buffer. The magnetic beads were incubated with anti-AGO2 antibody (cat. no. ab186733, Abcam) or anti-IgG antibody (cat. no. ab172730, Abcam) under rotation for 30 min at room temperature. The labeled magnetic beads were harvested, and 900 μl of RIP immunoprecipitation buffer and 100 μl of cell lysate were added to the labeled magnetic beads and incubated with rotation for 3 h overnight at 4 °C. RNeasy MinElute Cleanup kit (Qiagen, Inc.) was used to extract the immunoprecipitated RNA, and extracted RNAs were reverse transcribed and subjected to qPCR to detect the abundance of targeted circRNAs.

### GEO database reanalysis and prediction of miRNAs

The differentially expressed miRNA data in osteosarcoma were downloaded from the GEO database (https://www.ncbi.nlm.nih.gov/geo/query/acc.cgi?acc=GSE65071). The circRNA-miRNA interaction networks were predicted using circBank^24^, and the mRNA–miRNA interaction networks were predicted using TargetScan (http://www.targetscan.org/vert_71/) and miRDB^25^ according to the instructions of the websites. Overlapping miRNAs in all the mentioned databases or websites were selected for further research.

### Dual-luciferase reporter assay

The procedure was performed as previously described^26^. Wild-type or mutant circROCK1-E3/E4 and PTEN luciferase reporter plasmids (circROCK1-E3/E4 wt, circROCK1-E3/E4 mut, PTEN wt and PTEN mut) containing miR-532-5p target sites or substitution of target sites were synthesized by RiboBio Co. Ltd. (Ribobio, Guangzhou, China). MiR-532-5p mimics and the corresponding negative control miRNA (miR NC) were also synthesized by RiboBio Co. Ltd. (Ribobio). HOS and U2OS cells were seeded onto 24-well plates at a concentration of 3 × 10^4^ cells per well. The synthesized luciferase reporter plasmids and miR-532-5p mimics/miR NC were cotransfected into HOS and U2OS cells using Lipofectamine 3000 (Invitrogen). Forty-eight hours later, luciferase activity was measured with a Dual-Luciferase Reporter Assay System (Promega Corporation) according to the manufacturer’s instructions.

### In vivo nude mouse model

The procedure was performed as previously reported^16^. Female nude mice aged 4-5 weeks were purchased from the Animal Care and Use Committee of Dalian Medical University Ltd. (Dalian, China) and kept under sterile specific-pathogen-free (SPF) conditions. HOS cells (1 × 10^6^, mixed with Matrigel, BD Bioscience, 1:1) stably overexpressing circROCK1-E3/E4 and with the corresponding blank vector were injected subcutaneously or intravenously for the determination of tumor growth and lung metastasis. Formatted subcutaneous tumor nodes were monitored weekly, and lung metastatic nodes were monitored at week four by [^18^F]-fluorodeoxyglucose (^18^F-FDG) positron emission tomography/computed tomography (PET/CT) scanning. The formatted subcutaneous tumor nodes or metastatic lung nodes were harvested for further detection. This study was carried out in accordance with the Guide for the Care and Use of Laboratory Animals of the National Institutes of Health and was approved by the Institute Research Medical Ethics Committee of Central Hospital affiliated with Shenyang Medical College.

### Statistical analysis

Statistical analysis was performed by GraphPad Prism 7.0. All numerical data are presented as the mean ± SD for multiple samples with at least three replicates. Differences between groups were evaluated by Student’s t test or one-way analysis of variance, as appropriate. Overall survival differences between patients with high or low levels of circROCK1-E3/E4 expression were analyzed using the Kaplan–Meier method and log-rank test. ^*^p < 0.05, ^**^p < 0.01, and ^***^p < 0.001 were considered to indicate statistically significant differences.

## Results

### CircROCK1-E3/E4 is downregulated and associated with poor prognosis of patients with osteosarcoma

**Fig. 1:**
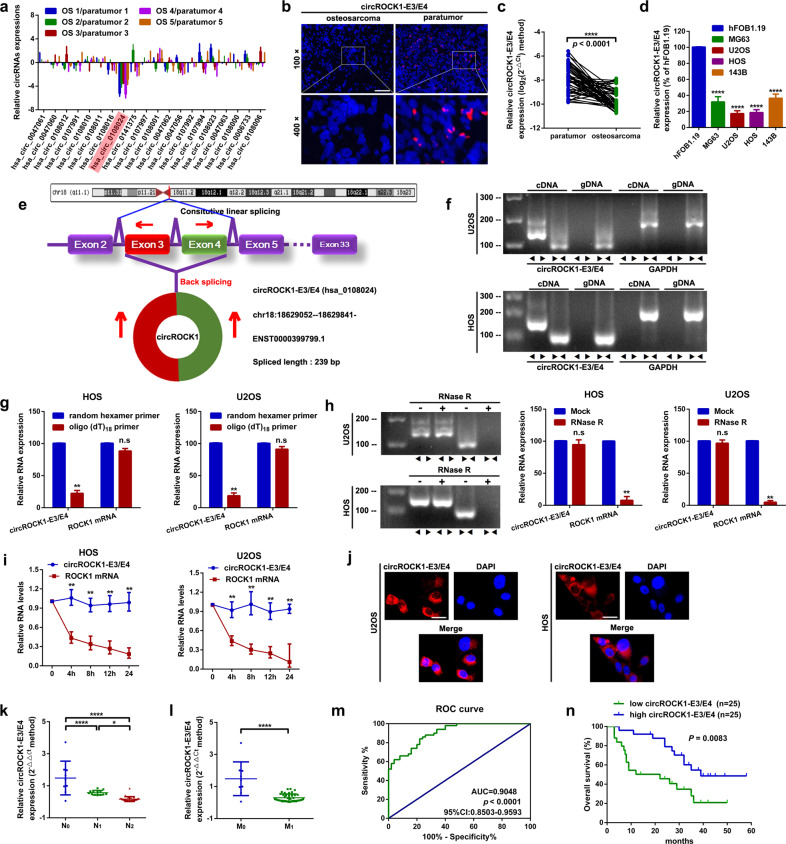
CircROCK1-E3/E4 is downregulated and associated with poor prognosis in patients with osteosarcoma. **a** The expression of certain ROCK1-derived circRNAs in osteosarcoma tissue specimens and paired paratumor tissue specimens was determined by an RT–qPCR assay. **b** Representative images of circROCK1-E3/E4 expression in osteosarcoma and paratumor tissues as displayed by a FISH assay. **c** The expression of circROCK1 in 50 osteosarcoma and paired paratumor tissue specimens was detected by RT–qPCR assay. ^****^p < 0.0001. **d** CircROCK1-E3/E4 was downregulated in osteosarcoma cell lines, as confirmed by an RT–qPCR assay. ^****^p < 0.0001. **e** CircROCK1-E3/E4 is circularized from exons 3 and 4 in ROCK1, as demonstrated by a schematic illustration. **f** CircROCK1-E3/E4 was amplified by divergent primers in cDNA but not gDNA by using GAPDH as a negative control, as validated by an RT–qPCR assay. **g** Compared with oligo (dT)_18_ primers, the expression of circROCK1-E3/E4 was significantly decreased when random hexamer primers were applied. ^n.s^*p* > 0.05 and ^**^p < 0.01. **h** Compared with linear ROCK1 mRNA, the expression changes of circROCK1-E3/E4 were unremarkable in the presence of RNase R. ^n.s^*p* > 0.05 and ^**^p < 0.01. **i** The RNA levels of circROCK1-E3/E4 and ROCK1 mRNA after 2 μg/ml actinomycin D treatment were quantified at different time points by qRT–PCR assays. ^**^p < 0.01. **j** CircROCK1-E3/E4 was mainly localized in the cytoplasm of U2OS and HOS cells. **k** CircROCK1-E3/E4 is frequently downregulated in patients with lymph node metastasis (N1 and N2). ^*^p < 0.05, ^****^p < 0.0001. **l** CircROCK1-E3/E4 is downregulated in patients with distant metastasis (M1 stage). ^****^p < 0.0001. **m** ROC curve analysis of the area under the curve (AUC) and 95% confidence interval (CI) of circROCK1-E3/E4 in patients with osteosarcoma. **n** A low expression level of circROCK1-E3/E4 was correlated with shorter survival rates in patients with osteosarcoma as determined by Kaplan–Meier analysis. All data are presented as the mean ± SD from three independent experiments.

It is widely accepted that most circRNAs are back-spliced from exons of their parental genes. In our previous research, we demonstrated that ROCK1 plays key roles in osteosarcoma progression^13–16,27^. In the current research, we focused on identifying potential circRNAs that might be derived from the ROCK1 gene. According to the online circRNA database circBase^18^, a total of 53 circRNAs are derived from the human ROCK1 gene (Supplementary Table 1 and Supplementary Fig. 1a). Among them, we chose 20 exonic circRNAs with lengths shorter than 500 bp for further detection in 5 osteosarcoma tissues and paired paratumor tissues. Based on the results of RT–qPCR displayed in Fig. 1a, hsa_circ_0108024, which we named circROCK1-E3/E4 because it was derived from exons 3 and 4 of the ROCK1 gene, was stably downregulated in the collected 5 osteosarcoma tissues. Through an RNA-FISH assay, we demonstrated that circROCK1-E3/E4 was downregulated in osteosarcoma tissues (Fig. 1b). Further RT–qPCR detection also indicated that circROCK1-E3/E4 was downregulated in osteosarcoma tissues (Fig. 1c). Additionally, we found that circROCK1-E3/E4 was downregulated in four osteosarcoma cell lines, MG63, U2OS, HOS and 143B, compared with that in the normal osteoblast cell line hFOB1.19 (Fig. 1d). By comparing the RNA sequence of its parental gene ROCK1 through analysis of circBase and the online circRNA annotation software circPrimer^28^, circROCK1-E3/E4 was back-spliced from exon 3 and exon 4 of the ROCK1 gene (Fig. 1e and Supplementary Fig. 1b). Furthermore, convergent and divergent primers were designed to verify the head-to-tail splicing model of circROCK1-E3/E4. As the results of nucleic acid electrophoresis displayed in Fig. 1f, circROCK1-E3/E4 could be detected in the cDNA of U2OS and HOS cells but not in gDNA. To further test the circular structure of circROCK1-E3/E4, a random hexamer primer and an oligo (dT)_18_ primer were reverse transcribed with RNAs extracted from U2OS and HOS cells. The expression of circROCK1-E3/E4 and ROCK1 mRNA was detected. As the results displayed in Fig. 1g show, compared with random hexamer primers, the expression of circROCK1-E3/E4 was aberrantly decreased in the oligo (dT)18 primer group. Meanwhile, the expression of ROCK1 mRNA changed insignificantly. This phenomenon suggested that circROCK1-E3/E4 had no poly-A tail. Next, we performed an RNase R assay and an actinomycin D assay to verify the stability of circROCK1-E3/E4, and the results are shown in Fig. 1h, i. circROCK1-E3/E4 was more stable than ROCK1 mRNA after RNase R and actinomycin D digestion. Furthermore, via an RNA-FISH assay, we confirmed that ROCK1-E3/E4 was mainly located in the cytoplasm of U2OS and HOS cells (Fig. 1j). Clinically, we demonstrated that circROCK1-E3/E4 was more frequently downregulated in osteosarcoma patients with lymph node metastasis and distant metastasis (Fig. 1k, l). Additionally, we applied ROC curve analysis to evaluate the clinical value of circROCK1-E3/E4. As presented in Fig. 1m, the area under the curve (AUC) was 0.9048 (with a 95% confidence interval of 0.8503–0.9593), indicating that circROCK1-E3/E4 might be a valuable prognostic biomarker for the diagnosis of osteosarcoma. According to the median value of circROCK1-E3/E4, the 50 collected cases were divided into a circROCK1-E3/E4 high group (*n* = 25) and a low group (*n* = 25). According to a Pearson chi-square test and Fisher’s exact test, we found that a low expression level of circROCK1-E3/E4 was closely correlated with advanced staging (IIB/III, *p* = 0.007) and distant metastasis (*p* = 0.023) and tumor size (*p* = 0.038) in patients with osteosarcoma (Table 2). Last, we demonstrated that a lower expression level of circROCK1-E3/E4 was intimately correlated with shorter overall survival rates in patients with osteosarcoma (*p* = 0.083) (Fig. 1n).

**Table 2:** Association of circROCK1-E3/E4 expression with clinicopathological features of osteosarcoma.

Features	No. of cases	circROCK1-E3/E4		*p*-value
		High	Low	
Age at diagnosis				1.000^a^
<18	37	18	19	
≥18	13	7	6	
Gender				1.000^a^
Female	29	14	15	
Male	21	11	10	
Histological subtype				1.000^a^
Osteoblastic	17	8	9	
Chondroblastic	10	5	5	
Fibroblastic	14	8	6	
Mixed	9	4	5	
Clinical stage				0.007^b^
I + IIA	18	14	4	
IIB/III	32	11	21	
Distant metastasis				0.023^b^
Absent	9	8	1	
Present	41	17	24	
Tumor size (cm)				0.038^a^
<5	18	13	5	
≥5	32	12	20	
Anatomic location				1.000^a^
Tibia/femur	37	19	18	
Elsewhere	13	6	7	

^a^*p*-value obtained from Pearson Chi-Square test.

^b^*p*-value obtained from Fisher’s Exact Test.

### CircROCK1-E3/E4 expression is partially regulated by QKI

**Fig. 2:**
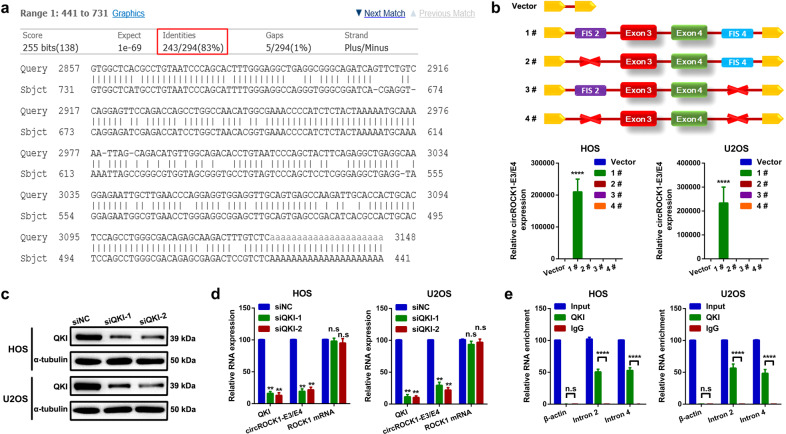
CircROCK1-E3/E4 expression is partially regulated by QKI. **a** The complementary sequences of intron 2 of ROCK1 were blasted with reverse sequences of intron 4 of ROCK1. Very highly similar sequences (83% identity over 294 nt) were found, as illustrated by using BLAST (https://blast.ncbi.nlm.nih.gov/Blast.cgi). **b** Four vectors (vector #1 containing FIS2 and FIS4; vector #2 with FIS2 deletion; vector #3 with FIS4 deletion; vector #4 with FIS2 and FIS4 deletion) were transfected into U2OS and HOS cells, and the expression level of circROCK1-E3/E4 was checked by an RT–qPCR assay. ^****^p < 0.0001. **c** QKI was knocked down as determined by a western blot assay. **d** Expression of QKI mRNA, circROCK1-E3/E4 and ROCK1 mRNA after transfection of QKI siRNAs was analyzed by an RT–qPCR assay. ^n.s^*p* > 0.05 and ^**^p < 0.01. **e** By using IgG as a control, partial segments of intron 2 and intron 4 of ROCK1 rather than β-actin were abundantly enriched in QKI, as measured by an RIP assay. ^n.s^*p* > 0.05 and ^****^p < 0.0001. All data are presented as the mean ± SD from three independent experiments.

It is widely accepted that circRNAs are usually generated from long flanking introns with inverted complementary sequences^29^. Therefore, we attempted to explore whether circROCK1-E3/E4 was formed via a similar mechanism. First, by blasting the complementary sequences of intron 2 and reverse sequences of intron 4 of ROCK1, we found very highly similar sequences (83% identity over 294 nt, Fig. 2a). The highly similar sequences in intron 2 of ROCK1 (position of Chr18: 18649177–18649468, hg19, which we named flanking intron segments 2, FIS2) and the sequence spanning from exon 3 of ROCK1 (position of Chr18: 18666972-18667072) to the highly similar sequences in intron 4 of ROCK1 (position of Chr18:18670654-18670944, hg19, which we named flanking intron segments 4, FIS4; in total of 3972 bp) were cloned into pZW1-snoVector^30^ (vector 1#). Moreover, different deletion constructs were cloned (vector 2# - 4#) separately (Fig. 2b, upper panel). All the constructed vectors were transfected into U2OS and HOS cells. Then, the expression levels of circROCK1-E3/E4 were quantified by qRT–PCR. As shown in Fig. 2b (lower panel), only vector #1 promoted circROCK1-E3/E4 expression in U2OS and HOS cells. It has been reported that the biogenesis of circRNAs is regulated by RNA-binding proteins (RBPs). Quaking (QKI), a well-known RBP, promotes circRNA formation by binding to its consensus target single-stranded RNA (ssRNA) motif in introns flanking circRNA-forming consensus target exons^31^. We attempted to explore whether QKI was also implicated in the biogenesis of circROCK1-E3/E4. We knocked down QKI via an RNAi technique (Fig. 2c) and assessed the expression of circROCK1-E3/E4 and ROCK1 mRNA by an RT–qPCR assay. As shown in Fig. 2d, knockdown of QKI suppressed the expression of circROCK1-E3/E4 but not ROCK1 mRNA. To confirm the targeted binding effect between QKI and sequences of intron 2 and intron 4, an RNA immunoprecipitation (RIP) assay was performed. As illustrated in Fig. 2e, compared with β-actin, partial sequences of intron 2 and intron 4 were abundantly enriched in QKI proteins rather than IgG.

#### CircROCK1-E3/E4 suppresses proliferation and migration in vitro

**Fig. 3:**
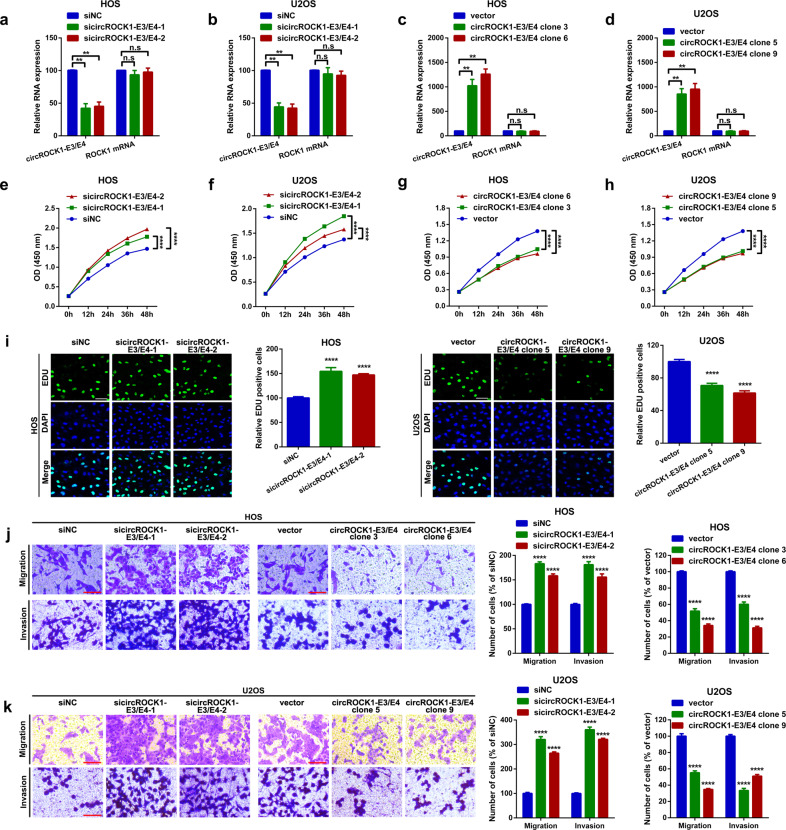
CircROCK1-E3/E4 suppresses osteosarcoma cell proliferation and migration in vitro. **a**, **b** CircROCK1-E3/E4 expression levels after transfection of specific siRNAs were measured by an RT–qPCR assay. ^n.s^*p* > 0.05 and ^**^p < 0.01. **c**, **d** Expression levels of circROCK1-E3/E4 and ROCK1 mRNA were analyzed by an RT–qPCR assay. ^n.s^*p* > 0.05 and ^**^p < 0.01. **e**–**h** Changes in cell *p*roliferation ability after various circROCK1-E3/E4 interventions were determined by a CCK8 assay. ^****^p < 0.0001. **i** An EdU assay was performed to evaluate changes in cell proliferation ability after up- and downregulation of circROCK1-E3/E4. ^****^p < 0.0001. **j**, **k** Cell motility ability changes after different circROCK1-E3/E4 interventions were determined by a transwell assay. ^****^p < 0.0001. All data are presented as the mean ± SD from three inde*p*endent experiments.

In this section, we attempted to assess the functional role of circROCK1-E3/E4 in osteosarcoma cells. We designed specific siRNAs targeting the back-splice sequence of circROCK1-E3/E4. As shown in Fig. 3a, b, circROCK1-E3/E4 was successfully knocked down, while a change in ROCK1 mRNA was not detected in U2OS and HOS cells. Additionally, the circROCK1-E3/E4 overexpression vector was utilized to upregulate the expression of circROCK1-E3/E4 in U2OS and HOS cells. As shown in Fig. 3c, d, circROCK1-E3/E4 was successfully upregulated in U2OS (clones 5 and 9) and HOS (clones 3 and 6) cells, while ROCK1 mRNA changed insignificantly. Subsequently, functional cell proliferation and motility assays were performed in the aforementioned cells. The CCK8 assay results shown in Fig. 3e, f revealed that downregulation of circROCK1-E3/E4 promoted proliferation in U2OS and HOS cells. In contrast, overexpression of circROCK1-E3/E4 suppressed the proliferation ability of U2OS and HOS cells (Fig. 3g, h). Additionally, the outcomes of the EdU assay indicated a similar tendency (Fig. 3i). Finally, a functional transwell chamber migration assay was performed to detect the migration ability changes in U2OS and HOS cells. As the representative photos displayed in Fig. 3j, k show, the downregulation of circROCK1-E3/E4 facilitated migration, while the overexpression of circROCK1-E3/E4 inhibited migration in U2OS and HOS cells.

#### CircROCK1-E3/E4 sponges miR-532-5p in U2OS and HOS cells

**Fig. 4:**
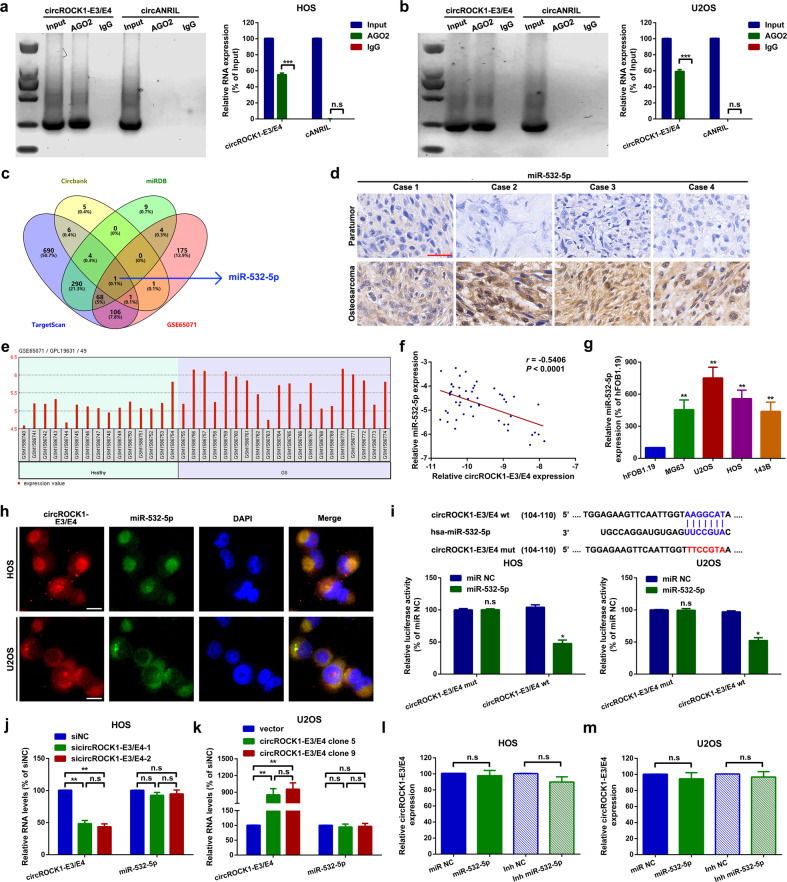
CircROCK1-E3/E4 sponges miR-532-5p in U2OS and HOS cells. **a**, **b** Compared with circANRIL, circROCK1 was abundantly enriched in AGO2 by using IgG as an internal control. ^n.s^*p* > 0.05 and ^***^p < 0.001 **c** A Venn diagram exhibiting overlapping miRNAs that might interact with circROCK1-E3/E4 and presented in GSE65071. **d** Expression of miR-532-5p in osteosarcoma tissues was illustrated by an in situ hybridization assay. **e** Expression of miR-532-5p in osteosarcoma tissues and paired healthy tissues in the osteosarcoma-related genome-wide study GSE65071. Probe ID 49 represents miR-532-5p. **f** Spearman correlation analysis showed that circROCK1-E3/E4 was inversely correlated with miR-532-5p. *r* = –0.5406 and *p* < 0.0001. **g** Expression levels of miR-532-5p in osteosarcoma cell lines were confirmed by an RT–qPCR assay. **h** The subcellular localization of circROCK1-E3/E4 and miR-532-5p was confirmed by a FISH assay. **i** The targeted binding effect and binding sites between circROCK1-E3/E4 and miR-532-5p were tested by a luciferase assay. ^n.s^*p* > 0.05 and ^*^p < 0.05. **j**, **k** Expression of miR-532-5p after up- and downregulation of circROCK1-E3/E4 was analyzed by RT–qPCR. ^n.s^*p* > 0.05 and ^**^p < 0.01. **l**, **m** Relative expression of circROCK1-E3/E4 after different miR-532-5p interventions was measured by RT–qPCR. ^n.s^*p* > 0.05. All data are presented as the mean ± SD from three independent experiments.

Accumulative evidence has indicated that cytoplasmic circRNAs can function as miRNA sponges^32^. Therefore, we attempted to explore whether circROCK1-E3/E4 works via a similar mechanism. First, using cANRIL (a circular RNA reported not to bind to Argonaute 2, AGO2) as a control, we performed a RIP assay to test whether the biological basis of circROCK1-E3/E4 serves as a miRNA sponge. As shown in Fig. 4a, b, compared with anti-IgG, circROCK1-E3/E4 rather than circANRIL was abundantly enriched in anti-AGO2. Furthermore, we used circBank^24^, miRDB^25^ and TargetScan^33^ as bioinformatics prediction tools to identify potential miRNAs that might interact with circROCK1-E3/E4 (Supplementary Tables 2 and 3). Among them, miR-532-5p was selected for further research, as it also overlapped in a previous osteosarcoma-related genome-wide study, GSE65071^34^ (Fig. 4c). We then quantified the expression level of miR-532-5p in osteosarcoma. According to an ISH assay, miR-532-5p was highly expressed in osteosarcoma tissues (Fig. 4d). Meanwhile, by using the online software GEO2R, it was revealed that miR-532-5p was upregulated in 20 osteosarcoma plasma samples by using 15 healthy controls (Fig. 4e, probe ID 49 represents miR-532-5p). A further Spearman correlation analysis indicated that circROCK1-E3/E4 was inversely correlated with miR-532-5p (Fig. 4f). Moreover, miR-532-5p was upregulated in 4 osteosarcoma cell lines compared with that in hFOB1.19 cells (Fig. 4g). Through a colocalized subcellular FISH assay, it was confirmed that circROCK1-E3/E4 and miR-532-5p were colocalized in the cytoplasm of U2OS and HOS cells (Fig. 4h). Furthermore, a luciferase assay was performed to confirm the targeted binding effects between circROCK1-E3/E4 and miR-532-5p. As illustrated in Fig. 4i, compared with negative control miRNA (miR NC), cotransfection of miR-532-5p mimics with wild circROCK1-E3/E4 reporter plasmids (circROCK1-E3/E4 wt) led to a significant decrease in luciferase activity. When mutant reporter plasmids (circROCK1-E3/E4 mut) were used, the luciferase activity was reinforced. Finally, through a series of qRT–PCR analyses, we found that up- and downregulation of circROCK1-E3/E4 had no obvious effect on miR-532-5p (Fig. 4j, k). On the other hand, the expression of circROCK1-E3/E4 was also not significantly influenced by miR-532-5p (Fig. 4l, m and Supplementary Fig. 2a, b).

#### MiR-532-5p promotes proliferation and migration by targeting PTEN in U2OS and HOS cells

**Fig. 5:**
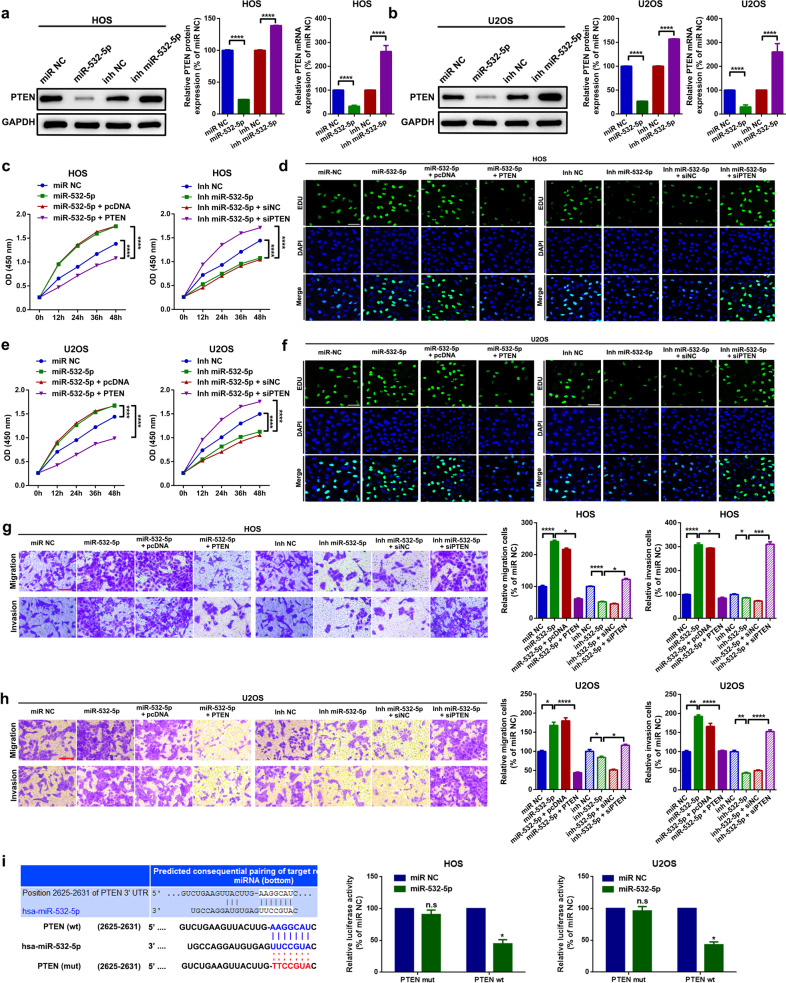
MiR-532-5p promotes proliferation and migration by targeting PTEN in U2OS and HOS cells. **a**, **b** Expression of PTEN after treatment with different miR-532-5p concentrations was determined by a western blot assay and an RT–qPCR assay. ^****^p < 0.0001. **c**–**f** Cell proliferation ability changes in HOS and U2OS cells were determined by CCK8 and EdU assays, respectively. ^****^p < 0.0001. **g**, **h** Cell migration and invasion ability changes in HOS and U2OS cells were measured by a transwell assay. ^*^p < 0.05, ^**^p < 0.01, ^***^p < 0.001, and ^****^p < 0.0001. **i** The targeted binding effect between miR-532-5p and PTEN was confirmed by a luciferase assay. The left panel shows the online prediction result by using TargetScan (http://www.targetscan.org/vert_72/), and the middle and right panels display the results of relative luminance changes. ^n.s^*p* > 0.05 and ^*^p < 0.05. All data are presented as the mean ± SD from three independent experiments.

As a well-known tumor suppressor, PTEN is extensively implicated in ncRNA-mediated cancer progression in multiple malignancies^35–38^. We first indicated that up- and downregulation of miR-532-5p inversely regulated PTEN expression (Fig. 5a, b). Functionally, as the data presented by the CCK8 assay and EdU assay displayed in Fig. 5c–f and Supplementary Fig. 3a–d, upregulation of miR-532-5p (transfection of miR-532-5p mimics) facilitated the proliferation of HOS and U2OS cells. This facilitative effect was significantly reversed by further upregulation of PTEN (cotransfection of miR-532-5p mimics and PTEN overexpression plasmids). In contrast, downregulation of miR-532-5p inhibited cell proliferation, and the suppressive effect was partially abolished by PTEN siRNA (cotransfection of miR-532-5p and siPTEN). Additionally, regarding the cell motility changes, the transwell assay indicated a similar tendency (Fig. 5g, h). Finally, through a luciferase assay, we confirmed that miR-532-5p targets PTEN by directly binding to the PTEN 3’UTR (Fig. 5i).

#### CircROCK1-E3/E4 suppresses proliferation and migration via regulation of the miR-532-5p/PTEN axis in U2OS and HOS cells

**Fig. 6:**
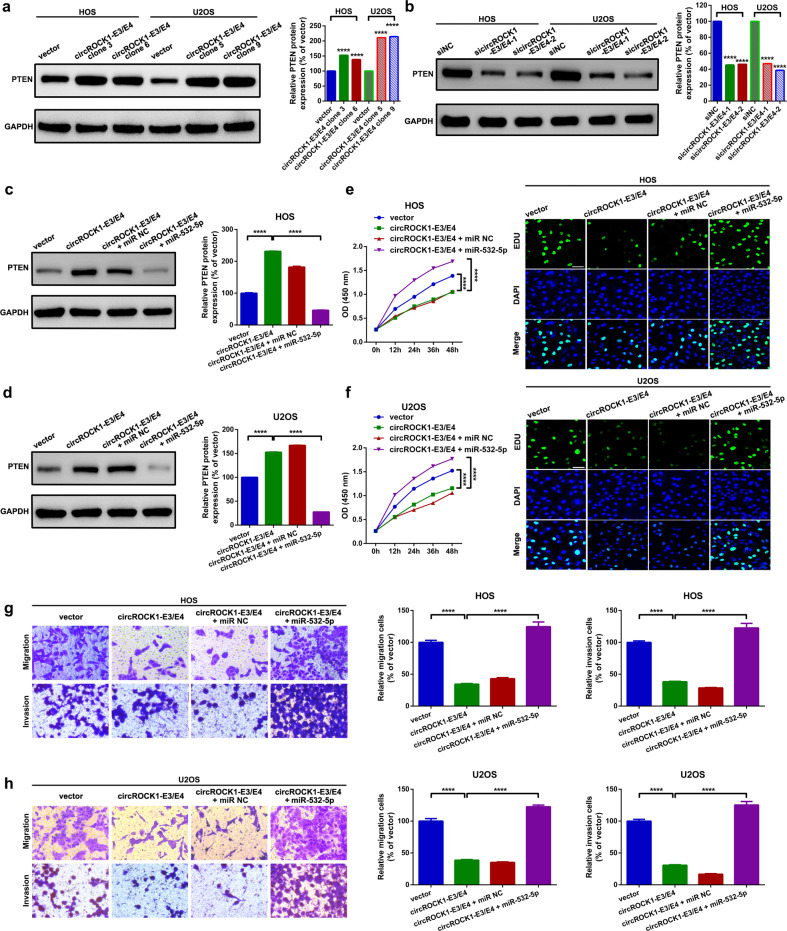
CircROCK1-E3/E4 suppresses proliferation and migration via regulation of the miR-532-5p/PTEN axis in U2OS and HOS cells. **a**, **b** Expression of PTEN protein was analyzed by western blot assay. ^****^p < 0.0001. **c**, **d** PTEN protein expression after different circROCK1-E3/E4 and miR-532-5p interventions was measured by western blot. ^****^p < 0.0001. **e**, **f** Cell proliferation ability changes were checked by a CCK8 assay and an EdU assay. ^****^p < 0.0001. **g**, **h** Cell motility changes were evaluated by a transwell assay. ^****^p < 0.0001. All data are presented as the mean ± SD from three independent experiments.

We finally asked whether circROCK1-E3/E4 suppresses proliferation and migration through the regulation of PTEN via miR-532-5p sponging. We first verified that circROCK1-E3/E4 positively regulated PTEN expression at the protein level (Fig. 6a, b). Subsequently, we focused on detecting the effect of circROCK1-E3/E4 and miR-532-5p on PTEN protein expression. As illustrated in Fig. 6c, d, circROCK1-E3/E4 promoted PTEN protein expression, while the promotive effect was reversed by miR-532-5p. Functionally, we found that the suppressive effects of circROCK1-E3/E4 on proliferation (Fig. 6e, f and Supplementary Fig. 4a, b) and migration/invasion (Fig. 6g, h) were abolished by miR-532-5p upregulation in HOS and U2OS cells. Taken together, these results indicated that circROCK1-E3/E4 promoted proliferation and migration/invasion, at least partially, via regulation of the miR-532-5p/PTEN axis.

#### CircROCK1-E3/E4 suppresses proliferation and lung metastasis in vivo

**Fig. 7:**
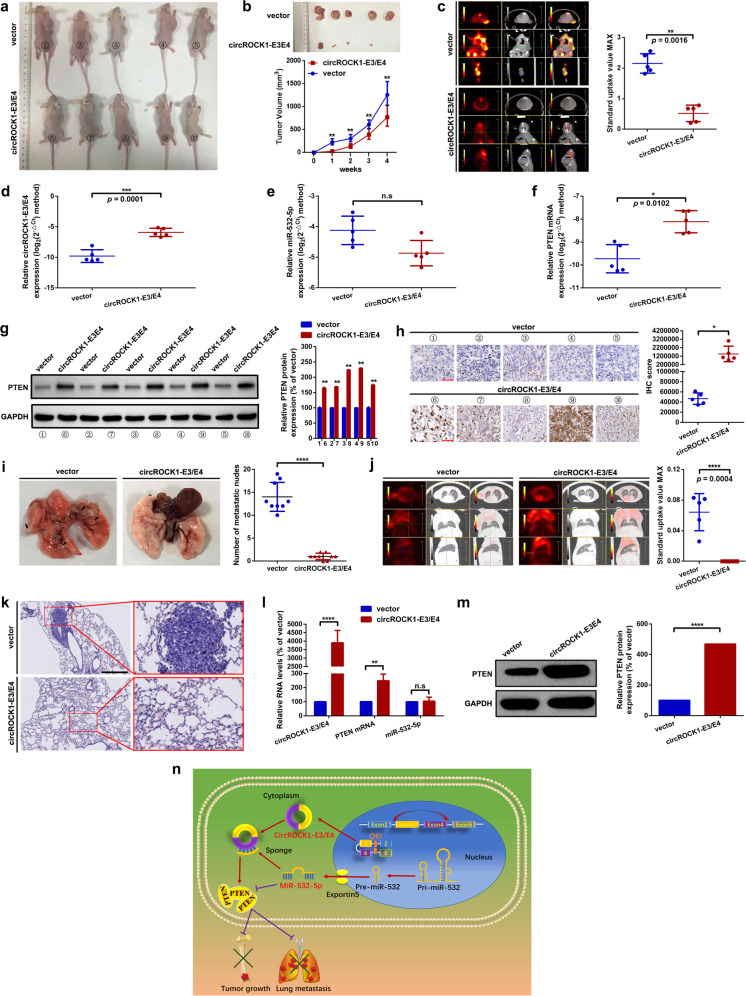
CircROCK1-E3/E4 suppresses proliferation and lung metastasis in vivo. **a** Macroscopic appearance of nude mice injected with osteosarcoma cells with stable overexpression of circROCK1-E3/E4 or with control cells (vector). **b** Overexpression of circROCK1-E3/E4 obviously restricted subcutaneous nodule formation. ^**^p < 0.01. **c** Representative ^18^F-FDG micro positron emission tomography/computed tomography (PET/CT) images of formatted subcutaneous nodes in mice with diverse circROCK1-E3/E4 expression. *p* = 0.0016. **d**–**f** Expression of circROCK1-E3/E4, miR-532-5p and PTEN mRNA in the formatted subcutaneous nodes was analyzed by RT–qPCR. ^n.s^*p* > 0.05, and *p*-values are presented in the figures accordingly. **g**, **h** PTEN protein expression in formatted subcutaneous nodes was determined by Western blot and IHC. ^*^p < 0.05 and ^**^p < 0^.^01. **i** Macroscopic appearance of formatted lung metastatic nodes in mice. ^****^p < 0.0001. **j** Representative ^18^F-FDG micro positron emission tomography/computed tomography (PET/CT) images of formatted lung metastatic nodes in mice with diverse circROCK1-E3/E4 expression. *p* = 0.0004. **k** H&E staining of formatted lung metastatic nodes; scale bars, 200 and 50 µm for magnifications of ×100 and ×400, respectively. **l** Expression levels of circROCK1-E3/E4, miR-532-5p, and PTEN mRNA in the formatted lung metastatic nodes were analyzed by RT–qPCR. ^n.s^*p* > 0.05, ^**^p < 0.01, and ^****^p < 0.0001. **m** PTEN protein expression in formatted lung metastatic nodes was determined by western blot. ^****^p < 0.0001. **n** Diagram indicating that circROCK1-E3/E4, back-spliced from exons 3 and exon 4 of the ROCK1 gene, which is regulated by QKI, suppressed cell proliferation and migration by altering the miR-532-5p/PTEN axis in osteosarcoma. All data are presented as the mean ± SD from three independent experiments.

To finally confirm the role of circROCK1-E3/E4 in tumor growth and lung metastasis, orthotopic xenograft mouse models were introduced. As the representative photographs displayed in Fig. 7a, b show, compared with the vector group, overexpression of circROCK1-E3/E4 significantly suppressed osteosarcoma tumor growth. Additionally, ^18^F-FDG PET/CT scans were used to assess the tumor growth of osteosarcoma in vivo. As the representative images show in Fig. 7c, the standard uptake values (SUVs) of the circROCK1-E3/E4 group were remarkably lower than those of the vector group (*p* = 0.0016). Furthermore, the expression of circROCK1-E3/E4, miR-532-5p and PTEN mRNA in the formatted subcutaneous nodes was determined by qRT–PCR assays. As shown in Fig. 7d–f, upregulated circROCK1-E3/E4 and PTEN mRNA were found in the circROCK1-E3/E4 group compared with the vector group. However, the expression changes of miR-532-5p were unremarkable. A further Western blot assay and IHC assay suggested that overexpression of circROCK1-E3/E4 promoted PTEN protein expression in the formatted subcutaneous nodes (Fig. 7g, h). Moreover, a pulmonary metastasis model of osteosarcoma in mice was constructed. As the representative photos demonstrate in Fig. 7i, overexpression of circROCK1-E3/E4 suppressed metastatic node formation in the lungs. Additionally, ^18^F-FDG PET/CT scans suggested that the SUVs of the circROCK1-E3/E4 group were significantly lower than those of the vector group (*p* = 0.0004) (Fig. 7j). Last, as illustrated in Fig. 7k–m, overexpression of circROCK1-E3/E4 promoted PTEN but had no effect on miR-532-5p expression in metastatic nodes in the lung. Together, these results confirmed that suppression of circROCK1-E3/E4 inhibited the lung metastasis of osteosarcoma in vivo.

## Discussion

Circular RNAs (circRNAs) are nonlinear conformation transcripts with scrambled exons but without polyadenylated (poly(A)) tails. Emerging studies have indicated that circRNAs are extensively implicated in various pathological processes, including neuronal function, innate immune responses, cell proliferation, and pluripotency^3^. Circular RNAs regulate their parental gene function by absorbing common miRNAs to increase miRNA availability or reduce free miRNA levels^6^. For example, circular RNA forkhead box O 3 (circ-Foxo3) and Foxo3 pseudogene (Foxo3P) sponge several miRNAs and increase Foxo3 translation^39^. In the current study, we focused on circular RNAs that might be back-spliced from the ROCK1 gene. We provided evidence that circROCK1-E3/E4, which is produced from exons 3 and 4 of ROCK1 precursor mRNA, was downregulated in osteosarcoma. Additionally, we found that downregulated circROCK1-E3/E4 was closely correlated with poor prognosis in patients with osteosarcoma. Mechanistically, we demonstrated that the introduction of circROCK1 was partially regulated by QKI, a well-known RBP belonging to the STAR family of KH domain-containing RBPs^40^.

The biogenesis of circRNAs can be regulated at different levels, including precursor RNA transcription, post- or cotranscriptional processing and turnover^3^. The QKI protein contains multiple proline-rich putative Src-homology 3 (SH3)-binding motifs, and QKI is implicated in RNA metabolism by binding to signaling proteins that contain SH3 domains^41^. As a classic nuclear RNA-binding protein, QKI regulates the formation of circRNAs by binding sites in introns flanking circRNA-forming exons^31^. In hepatocellular carcinoma (HCC), knockdown of QKI significantly reduced circular RNA zinc-finger protein with KRAB and SCAN domains 1 (circZKSCAN1) expression in HCC cell lines SMMC7721 and HCC-LM3^42^. In this study, we provided proof that QKI promoted circROCK1-E3/E4 formation posttranscriptionally by binding to flanking sequences (intron 2 and intron 4) of ROCK1 pre-mRNA.

CircRNAs function via multiple mechanisms, such as serving as miRNA sponges, interacting with proteins, being translated into proteins and regulating transcription^3^. In the present research, by functional in vivo and in vitro assays, we found that circROCK1-E3/E4 promoted proliferation and migration by sequestering miR-532-5p, thereby promoting PTEN-mediated proliferation and migration in osteosarcoma. MiRNAs are extensively implicated in multiple aspects of cancer progression, such as proliferation, apoptosis, angiogenesis, migration, invasion, drug resistance and EMT^43–47^. The functional role of miR-532-5p in cancer remains controversial. MiR-532-5p serves as an oncogene in various malignancies, such as colorectal cancer, lung cancer, ovarian cancer and renal cancer^48–51^. Furthermore, it functions as an oncogenic microRNA and promotes gastric cancer cell proliferation and migration^52^. The current research first illustrates the role of miR-532-5p in osteosarcoma. Although the mechanism of ceRNAs in the cancer field is no longer a trending topic, our present study still provides novel evidence of miR-532-5p in osteosarcoma. We found that miR-532-5p can act as a bridge between circROCK1/E3E4 and PTEN. Additionally, our results indicated that both circROCK1/E3E4 and PTEN were targets of miR-532-5p.

PTEN, a well-known tumor suppressor, is widely involved in the progression of various cancers, including cell metabolism, cell motility, cell polarity and the tumor microenvironment^53^. It negatively regulates the phosphatidylinositol 3-kinase (PI3K)/protein kinase B (AKT)/mammalian target of rapamycin (mTOR) pathway in osteosarcoma. Our present work showed that PTEN serves as a downstream target of miR-532-5p and is closely implicated in cell proliferation and migration in osteosarcoma.

In conclusion, as shown in the diagram displayed in Fig. 7n, our findings in the present research are the first to reveal that circROCK1/E3E4, regulated by QKI, suppressed cell proliferation and migration by altering the miR-532-5p/PTEN axis in osteosarcoma. Our findings might lay the foundation for further functional, diagnostic and therapeutic research of circRNAs in osteosarcoma.

## Supplementary information

Supplementary materials

## References

[1] Philip T (2001). Osteosarcoma. Br. J. Cancer.

[2] Ottaviani G, Jaffe N (2009). The epidemiology of osteosarcoma. Cancer Treat. Res..

[3] Li X, Yang L, Chen LL (2018). The Biogenesis, Functions, and Challenges of Circular RNAs. Mol. Cell.

[4] Memczak S (2013). Circular RNAs are a large class of animal RNAs with regulatory potency. Nature.

[5] Hansen TB (2013). Natural RNA circles function as efficient microRNA sponges. Nature.

[6] An Y, Furber KL, Ji S (2017). Pseudogenes regulate parental gene expression via ceRNA network. J. Cell. Mol. Med..

[7] Zhang Y, Li J, Wang Y, Jing J, Li J (2019). The roles of circular RNAs in osteosarcoma. Med. Sci. Monit..

[8] Wang C, Ren M, Zhao X, Wang A, Wang J (2018). Emerging roles of circular RNAs in osteosarcoma. Med. Sci. Monit..

[9] Wu Y (2019). Circular RNA circTADA2A promotes osteosarcoma progression and metastasis by sponging miR-203a-3p and regulating CREB3 expression. Mol. Cancer.

[10] Schackmann RC (2011). Cytosolic p120-catenin regulates growth of metastatic lobular carcinoma through Rock1-mediated anoikis resistance. J. Clin. Invest..

[11] Nakagawa O (1996). ROCK-I and ROCK-II, two isoforms of Rho-associated coiled-coil forming protein serine/threonine kinase in mice. FEBS Lett..

[12] Wei L, Surma M, Shi S, Lambert-Cheatham N, Shi J (2016). Novel insights into the roles of Rho kinase in cancer. Arch. Immunol. Ther. Exp..

[13] Wang Y, Zhao W, Fu Q (2013). miR-335 suppresses migration and invasion by targeting ROCK1 in osteosarcoma cells. Mol. Cell. Biochem..

[14] Wang Y (2017). MicroRNA-335 and its target Rock1 synergistically influence tumor progression and prognosis in osteosarcoma. Oncol. Lett..

[15] Wang Y (2017). Long non-coding RNA TUG1 promotes migration and invasion by acting as a ceRNA of miR-335-5p in osteosarcoma cells. Cancer Sci..

[16] Wang Y (2018). Long noncoding RNA DANCR, working as a competitive endogenous RNA, promotes ROCK1-mediated proliferation and metastasis via decoying of miR-335-5p and miR-1972 in osteosarcoma. Mol. Cancer.

[17] Salzman J, Chen RE, Olsen MN, Wang PL, Brown PO (2013). Cell-type specific features of circular RNA expression. PLoS Genet..

[18] Glažar P, Papavasileiou P, Rajewsky N (2014). circBase: a database for circular RNAs. RNA.

[19] Rybak-Wolf A (2015). Circular RNAs in the mammalian brain are highly abundant, conserved, and dynamically expressed. Mol. Cell.

[20] Cheng Z (2019). circTP63 functions as a ceRNA to promote lung squamous cell carcinoma progression by upregulating FOXM1. Nat. Commun..

[21] Zeng K (2018). CircHIPK3 promotes colorectal cancer growth and metastasis by sponging miR-7. Cell Death Dis..

[22] Kocks C, Boltengagen A, Piwecka M, Rybak-Wolf A, Rajewsky N (2018). Single-molecule fluorescence in situ hybridization (FISH) of circular RNA CDR1as. Methods Mol. Biol..

[23] Nan A (2019). Circular RNA circNOL10 Inhibits Lung Cancer Development by Promoting SCLM1-Mediated Transcriptional Regulation of the Humanin Polypeptide Family. Adv. Sci. (Weinh.).

[24] Liu M, Wang Q, Shen J, Yang BB, Ding X (2019). Circbank: a comprehensive database for circRNA with standard nomenclature. RNA Biol..

[25] Chen Y, Wang X (2020). miRDB: an online database for prediction of functional microRNA targets. Nucleic Acids Res..

[26] Chen Z (2020). Circular RNA hsa_circ_001895 serves as a sponge of microRNA-296-5p to promote clear cell renal cell carcinoma progression by regulating SOX12. Cancer Sci..

[27] Wang Y (2017). Long non-coding RNA MALAT1 for promoting metastasis and proliferation by acting as a ceRNA of miR-144-3p in osteosarcoma cells. Oncotarget.

[28] Zhong S, Wang J, Zhang Q, Xu H, Feng J (2018). CircPrimer: a software for annotating circRNAs and determining the specificity of circRNA primers. BMC Bioinforma..

[29] Zhang XO (2014). Complementary sequence-mediated exon circularization. Cell.

[30] Yin QF (2015). SnoVectors for nuclear expression of RNA. Nucleic Acids Res..

[31] Conn SJ (2015). The RNA binding protein quaking regulates formation of circRNAs. Cell.

[32] Zhong Y (2018). Circular RNAs function as ceRNAs to regulate and control human cancer progression. Mol. Cancer.

[33] Agarwal V, Bell GW, Nam JW, Bartel DP (2015). Predicting effective microRNA target sites in mammalian mRNAs. eLife.

[34] Allen-Rhoades W (2015). Cross-species identification of a plasma microRNA signature for detection, therapeutic monitoring, and prognosis in osteosarcoma. Cancer Med..

[35] Xi Y, Chen Y (2015). Oncogenic and therapeutic targeting of PTEN loss in bone malignancies. J. Cell. Biochem..

[36] Poliseno L, Pandolfi PP (2015). PTEN ceRNA networks in human cancer. Methods.

[37] Li W, Zhang T, Guo L, Huang L (2018). Regulation of PTEN expression by noncoding RNAs. J. Exp. Clin. Cancer Res..

[38] Ghafouri-Fard S (2021). Regulatory role of microRNAs on PTEN signaling. Biomed. Pharmacother..

[39] Yang W, Du WW, Li X, Yee AJ, Yang BB (2016). Foxo3 activity promoted by non-coding effects of circular RNA and Foxo3 pseudogene in the inhibition of tumor growth and angiogenesis. Oncogene.

[40] Hall MP (2013). Quaking and PTB control overlapping splicing regulatory networks during muscle cell differentiation. RNA.

[41] Zhang Y (2003). Tyrosine phosphorylation of QKI mediates developmental signals to regulate mRNA metabolism. EMBO J..

[42] Zhu YJ (2019). Circular RNAs negatively regulate cancer stem cells by physically binding FMRP against CCAR1 complex in hepatocellular carcinoma. Theranostics.

[43] Gomes BC, Rueff J, Rodrigues AS (2016). MicroRNAs and cancer drug resistance. Methods Mol. Biol..

[44] Lee YS, Dutta A (2009). MicroRNAs in cancer. Annu. Rev. Pathol..

[45] Lou W (2017). MicroRNAs in cancer metastasis and angiogenesis. Oncotarget.

[46] Zaravinos A (2015). The regulatory role of MicroRNAs in EMT and cancer. J. Oncol..

[47] Si W, Shen J, Zheng H, Fan W (2019). The role and mechanisms of action of microRNAs in cancer drug resistance. Clin. Epigenet..

[48] Bjeije H, Soltani BM, Behmanesh M, Zali MR (2019). YWHAE long non-coding RNA competes with miR-323a-3p and miR-532-5p through activating K-Ras/Erk1/2 and PI3K/Akt signaling pathways in HCT116 cells. Hum. Mol. Genet..

[49] Zhai W (2018). MiR-532-5p suppresses renal cancer cell proliferation by disrupting the ETS1-mediated positive feedback loop with the KRAS-NAP1L1/P-ERK axis. Br. J. Cancer.

[50] Hu J, Wang L, Guan C (2020). MiR-532-5p suppresses migration and invasion of lung cancer cells through inhibiting CCR4. Cancer Biother. Radiopharm..

[51] Wei H, Tang QL, Zhang K, Sun JJ, Ding RF (2018). miR-532-5p is a prognostic marker and suppresses cells proliferation and invasion by targeting TWIST1 in epithelial ovarian cancer. Eur. Rev. Med. Pharmacol. Sci..

[52] Xu X, Zhang Y, Liu Z, Zhang X, Jia J (2016). miRNA-532-5p functions as an oncogenic microRNA in human gastric cancer by directly targeting RUNX3. J. Cell. Mol. Med..

[53] Song MS, Salmena L, Pandolfi PP (2012). The functions and regulation of the PTEN tumour suppressor. Nat. Rev. Mol. Cell Biol..

