# Supplementary material for: Circular RNA ROCK1, a novel circRNA, suppresses osteosarcoma proliferation and migration via altering the miR-532-5p/PTEN axis
Source: Exp Mol Med. 2022 Jul 25;54(7):1024–37. doi: 10.1038/s12276-022-00806-z (PMC9356001; doi:10.1038/s12276-022-00806-z)
Supplement: Supplementary file 1 — Supplementary materials [file 12276_2022_806_MOESM1_ESM.pdf]

Supplementary Materials

Circular RNA ROCK1, a novel circRNA, suppresses osteosarcoma proliferation and migration via altering the miR-532-5p/PTEN axis

Yize Liu[1,2], Guanzhen Qiu[2], Yinzhou Luo[2], Shanshan Li[3], Yeqiu Xu[2], Yuanzhuang Zhang[2], Jiayuan Hu[4], Peifeng Li[5], Hai Pan[6], Yong Wang[1,2,7*]

Supplementary Figures

Supplementary Fig.1

a

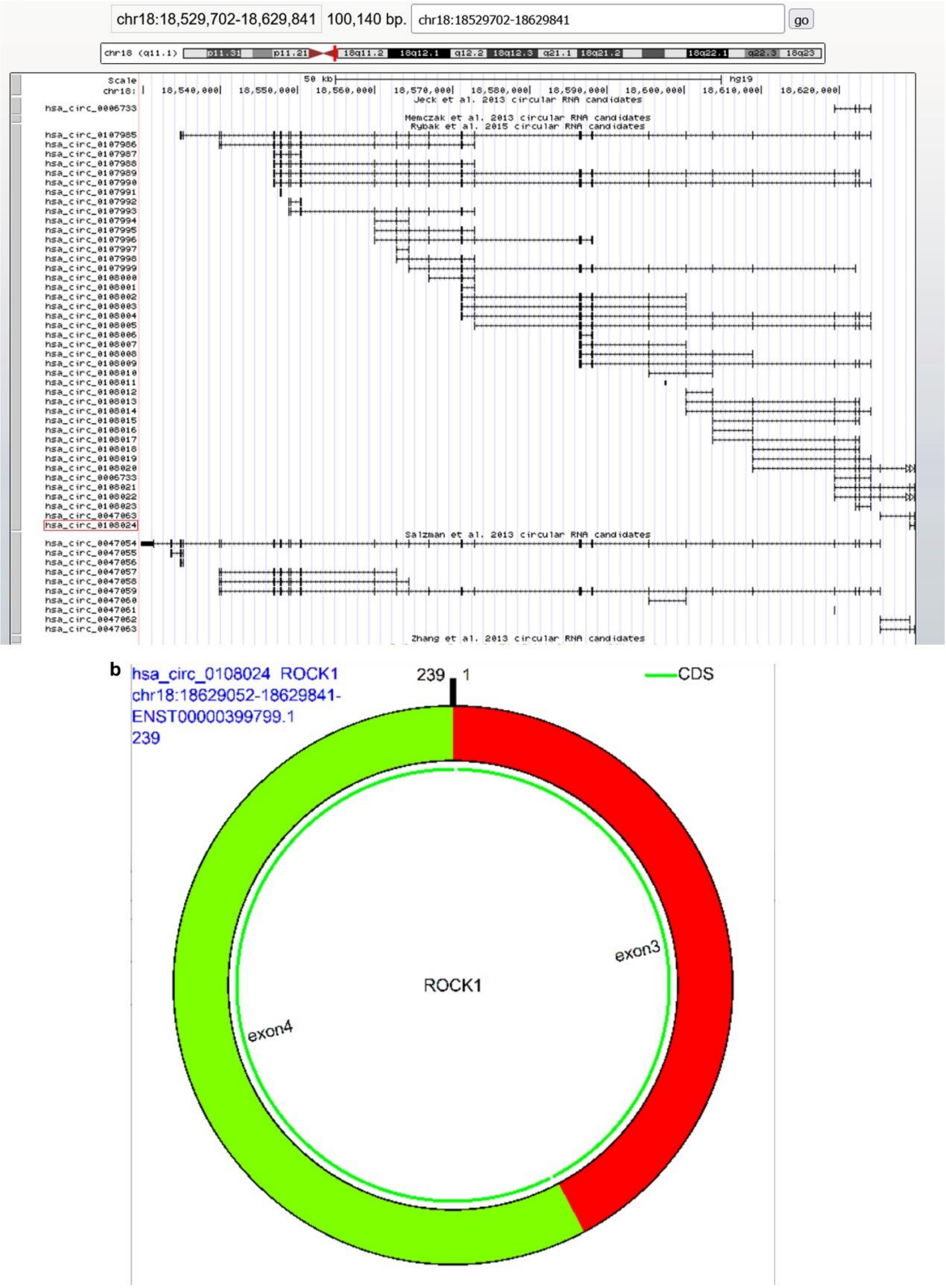

Supplementary Fig.1 (a) CircRNAss that might be derived from ROCK1 gene, as illustrated in UCSC Genome Browser. (b) CircROCK1-E3/E4 (hsa_circ_0108024) is

back-spliced from exon 3 and exon 4 of ROCK1 gene as indicated by circPrimer.

Supplementary Fig.2

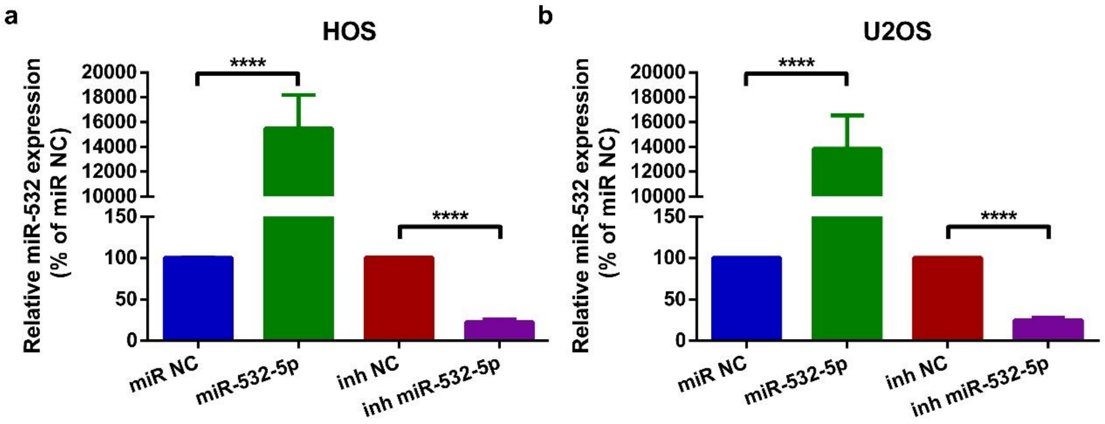

Supplementary Fig.2 (a-b) The expression of miR-532-5p after transfection of miR-532-5p mimics or inhibitors in HOS and U2OS cells were confirmed by a RT-qPCR assays. $^{****}p < 0.0001$. All data are presented as the mean ± SD from three independent experiments.

Supplementary Fig.3

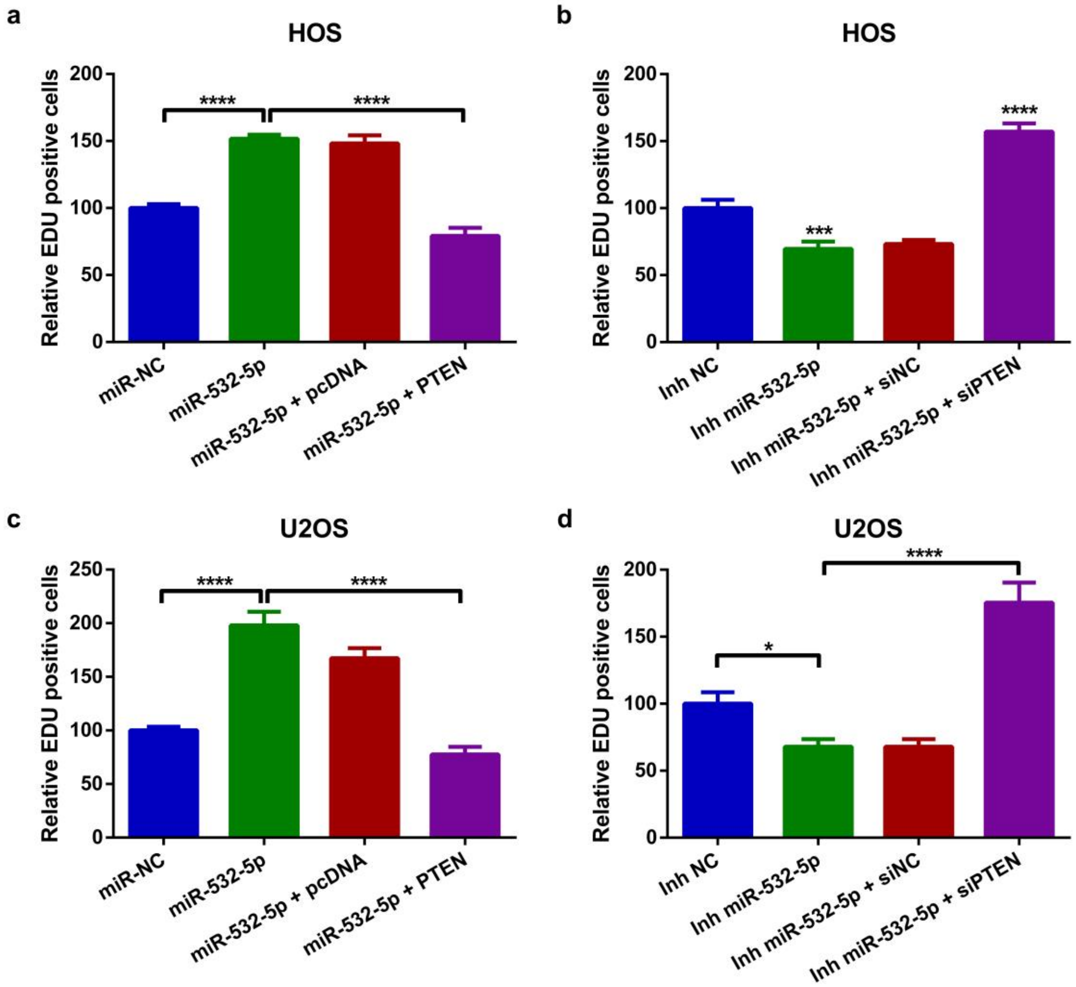

Supplementary Fig.3 (a-d) The cell proliferation ability changes of HOS (a and b) and U2OS (c and d) cells after transfection of diverse miR-532-5p oligonucleotides or specific PTEN siRNAs were qualified by an EDU assay. $^{****}p < 0.0001$. All data are presented as the mean ± SD from three independent experiments.

Supplementary Fig.4

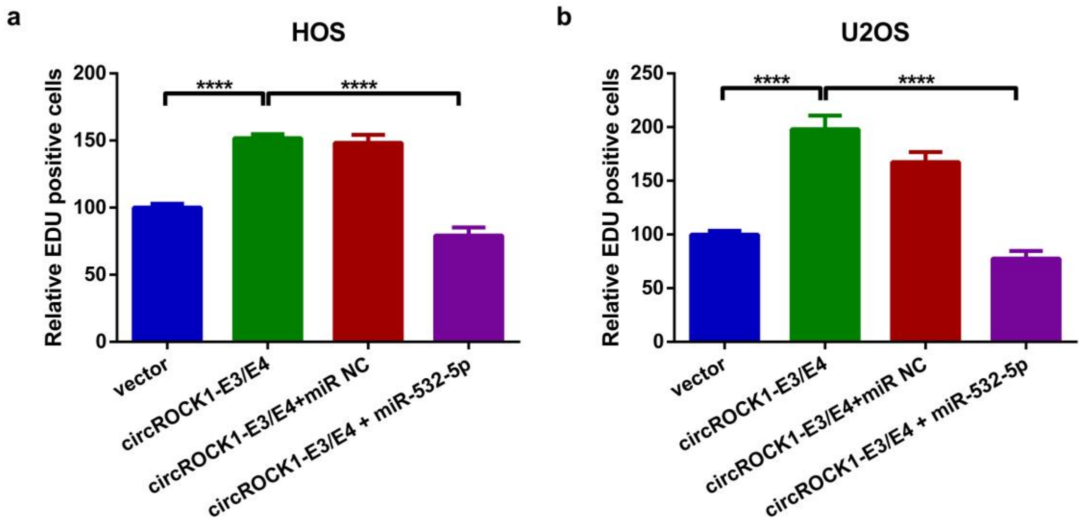

Supplementary Fig.4 (a-b) The cell proliferation ability changes of HOS and U2OS cells after different circROCK1-E3/E4 or miR-532-5p intervene were determiend by an EDU assay. $^{****}p < 0.0001$. All data are presented as the mean ± SD from three independent experiments.

Supplementary Tables

Supplementary Table 1. CircRNAs derived from human ROCK1 gene

Position	CircRNA ID	Genomic length	Spliced length	Best transcript	Gene symbol	CircRNA study
chr18:18619432-18619524	hsa_circ_0047061	92	92	NM_005406	ROCK1	Salzman2013
chr18:18595391-18600200	hsa_circ_0047060	4809	138	NM_005406	ROCK1	Salzman2013
chr18:18600111-18603641	hsa_circ_0108012	3530	150	ENST00000399799.1	ROCK1	Rybak2015
chr18:18547712-18547901	hsa_circ_0107991	189	189	ENST00000399799.1	ROCK1	Rybak2015
chr18:18595391-18603641	hsa_circ_0108010	8250	199	ENST00000399799.1	ROCK1	Rybak2015
chr18:18597458-18597673	hsa_circ_0108011	215	215	ENST00000399799.1	ROCK1	Rybak2015
chr18:18603580-18608896	hsa_circ_0108016	5316	221	ENST00000399799.1	ROCK1	Rybak2015
chr18:18629052-18629841	hsa_circ_0108024	789	239	ENST00000399799.1	ROCK1	Rybak2015
chr18:18562723-18564496	hsa_circ_0141375	1773	255	ENST00000399799.1	ROCK1	Maass2017
chr18:18562723-18564496	hsa_circ_0107997	1773	255	ENST00000399799.1	ROCK1	Rybak2015
chr18:18571136-18572898	hsa_circ_0108001	1762	258	ENST00000399799.1	ROCK1	Rybak2015

chr18:18625252-18629190	hsa_circ_0047062	3938	314	NM_005406	ROCK1	Salzman2013
chr18:18534743-18535206	hsa_circ_0047056	463	341	NM_005406	ROCK1	Salzman2013
chr18:18548732-18550474	hsa_circ_0107992	1742	349	ENST00000399799.1	ROCK1	Rybak2015
chr18:18559870-18564496	hsa_circ_0107994	4626	350	ENST00000399799.1	ROCK1	Rybak2015
chr18:18622057-18624147	hsa_circ_0108023	2090	369	ENST00000399799.1	ROCK1	Rybak2015
chr18:18625252-18629841	hsa_circ_0047063	4589	415	NM_005406	ROCK1	Rybak2015, Salzman2013
chr18:18566910-18572898	hsa_circ_0108000	5988	419	ENST00000399799.1	ROCK1	Rybak2015
chr18:18619432-18624147	hsa_circ_0006733	4715	461	NM_005406	ROCK1	Jeck2013, Maass2017, Rybak2015
chr18:18586311-18588155	hsa_circ_0108006	1844	475	ENST00000399799.1	ROCK1	Rybak2015
chr18:18608736-18622670	hsa_circ_0108018	13934	536	ENST00000399799.1	ROCK1	Rybak2015
chr18:18533538-18535206	hsa_circ_0047055	1668	549	NM_005406	ROCK1	Salzman2013
chr18:18603576-18622666	hsa_circ_0108015	19090	597	ENST00000399799.1	ROCK1	Rybak2015
chr18:18603580-18622670	hsa_circ_0108017	19090	597	ENST00000399799.1	ROCK1	Rybak2015
chr18:18586311-18600200	hsa_circ_0108007	13889	613	ENST00000399799.1	ROCK1	Rybak2015

chr18:18608736-18624147	hsa_circ_0108019	15411	621	ENST00000399799.1	ROCK1	Rybak2015
chr18:18562723-18572898	hsa_circ_0107998	10175	674	ENST00000399799.1	ROCK1	Rybak2015
chr18:18600111-18622670	hsa_circ_0108013	22559	686	ENST00000399799.1	ROCK1	Rybak2015
chr18:18546877-18550474	hsa_circ_0107987	3597	698	ENST00000399799.1	ROCK1	Rybak2015
chr18:18559870-18572898	hsa_circ_0107995	13028	769	ENST00000399799.1	ROCK1	Rybak2015
chr18:18559870-18572898	hsa_circ_0141374	13028	769	ENST00000399799.1	ROCK1	Maass2017
chr18:18600111-18624147	hsa_circ_0108014	24036	771	ENST00000399799.1	ROCK1	Rybak2015
chr18:18586311-18608896	hsa_circ_0108008	22585	834	ENST00000399799.1	ROCK1	Rybak2015
chr18:18571136-18600197	hsa_circ_0108002	29061	868	ENST00000399799.1	ROCK1	Rybak2015
chr18:18571136-18600200	hsa_circ_0108003	29064	871	ENST00000399799.1	ROCK1	Rybak2015
chr18:18619432-18629841	hsa_circ_0108021	10409	876	ENST00000399799.1	ROCK1	Rybak2015
chr18:18619432-18650574	hsa_circ_0108022	31142	958	ENST00000399799.1	ROCK1	Rybak2015
chr18:18539800-18562793	hsa_circ_0047057	22993	1023	NM_005406	ROCK1	Salzman2013
chr18:18608736-18650574	hsa_circ_0108020	41838	1118	ENST00000399799.1	ROCK1	Rybak2015

chr18:18548732-18572898	hsa_circ_0107993	24166	1118	ENST00000399799.1	ROCK1	Rybak2015
chr18:18539800-18564496	hsa_circ_0047058	24696	1208	NM_005406	ROCK1	Salzman2013
chr18:18559870-18588155	hsa_circ_0107996	28285	1244	ENST00000399799.1	ROCK1	Rybak2015
chr18:18586311-18624147	hsa_circ_0108009	37836	1295	ENST00000399799.1	ROCK1	Rybak2015
chr18:18572791-18624147	hsa_circ_0108005	51356	1402	ENST00000399799.1	ROCK1	Rybak2015
chr18:18546877-18572898	hsa_circ_0107988	26021	1467	ENST00000399799.1	ROCK1	Rybak2015
chr18:18571136-18624147	hsa_circ_0108004	53011	1553	ENST00000399799.1	ROCK1	Rybak2015
chr18:18539800-18572898	hsa_circ_0107986	33098	1627	ENST00000399799.1	ROCK1	Rybak2015
chr18:18564311-18622196	hsa_circ_0107999	57885	1669	ENST00000399799.1	ROCK1	Rybak2015
chr18:18546877-18622670	hsa_circ_0107989	75793	2677	ENST00000399799.1	ROCK1	Rybak2015
chr18:18546877-18624147	hsa_circ_0107990	77270	2762	ENST00000399799.1	ROCK1	Rybak2015
chr18:18539800-18625428	hsa_circ_0047059	85628	3098	NM_005406	ROCK1	Salzman2013
chr18:18534743-18624147	hsa_circ_0107985	89404	3263	ENST00000399799.1	ROCK1	Rybak2015
chr18:18529702-18625428	hsa_circ_0047054	95726	5293	NM_005406	ROCK1	Salzman2013

Supplementary Table 2. Potential miRNAs that might be interacted with circROCK1-E3/E4 (hsa_circ_0108024) as predicted by circBank

circBank ID	circbase ID	length	miRNA ID (miR_ID)	miRanda binding site (positions)	targetscan binding site (positions)
hsa_circROCK1_003	hsa_circ_0108024	239	hsa-miR-548e-5p	164	179-185, 210-215
hsa_circROCK1_003	hsa_circ_0108024	239	hsa-miR-1298-5p	18	33-40
hsa_circROCK1_003	hsa_circ_0108024	239	hsa-miR-138-1-3p	39	54-60
hsa_circROCK1_003	hsa_circ_0108024	239	hsa-miR-204-5p	184	196-202
hsa_circROCK1_003	hsa_circ_0108024	239	hsa-miR-29a-5p	4	19-26
hsa_circROCK1_003	hsa_circ_0108024	239	hsa-miR-3144-3p	110	124-130
hsa_circROCK1_003	hsa_circ_0108024	239	hsa-miR-3169	210	221-227
hsa_circROCK1_003	hsa_circ_0108024	239	hsa-miR-4633-5p	93	105-112
hsa_circROCK1_003	hsa_circ_0108024	239	hsa-miR-4659b-3p	176	192-198
hsa_circROCK1_003	hsa_circ_0108024	239	hsa-miR-4705	54	66-72
hsa_circROCK1_003	hsa_circ_0108024	239	hsa-miR-4724-3p	108	122-128
hsa_circROCK1_003	hsa_circ_0108024	239	hsa-miR-515-5p	70	85-92
hsa_circROCK1_003	hsa_circ_0108024	239	hsa-miR-532-5p	90	104-111
hsa_circROCK1_003	hsa_circ_0108024	239	hsa-miR-548aj-5p	194	211-217
hsa_circROCK1_003	hsa_circ_0108024	239	hsa-miR-548f-5p	197	211-217

hsa_circROCK1_003	hsa_circ_0108024	239	hsa-miR-548g-5p	194	211-217
hsa_circROCK1_003	hsa_circ_0108024	239	hsa-miR-548x-5p	194	211-217
hsa_circROCK1_003	hsa_circ_0108024	239	hsa-miR-6834-3p	187	199-205

Supplementary Table 3. Potential miRNAs that might be interacted with circROCK1-E3/E4 (hsa_circ_0108024) as predicted by miRDB

Target Rank	Target Score	miRNA Name	Gene Symbol
1	83	hsa-miR-1298-5p	hsa_circ_0108024
2	74	hsa-miR-29a-5p	hsa_circ_0108024
3	64	hsa-miR-3165	hsa_circ_0108024
4	60	hsa-miR-519e-5p	hsa_circ_0108024
5	60	hsa-miR-515-5p	hsa_circ_0108024
6	60	hsa-miR-607	hsa_circ_0108024
7	59	hsa-miR-138-1-3p	hsa_circ_0108024
8	58	hsa-miR-6814-5p	hsa_circ_0108024
9	56	hsa-miR-532-5p	hsa_circ_0108024

10	56	hsa-miR-219b-5p	hsa_circ_0108024
11	53	hsa-miR-216b-5p	hsa_circ_0108024
12	52	hsa-miR-4456	hsa_circ_0108024
